# From Free Binding Energy Calculations of SARS-CoV-2—Receptor Interactions to Cellular Immune Responses

**DOI:** 10.3390/medicina58020226

**Published:** 2022-02-02

**Authors:** Michael O. Glocker, Kwabena F. M. Opuni, Hans-Juergen Thiesen

**Affiliations:** 1 Proteome Center Rostock, University Medicine Rostock, University of Rostock, Schillingallee 69, 18059 Rostock, Germany; 2 Department of Pharmaceutical Chemistry, School of Pharmacy, College of Health Science, University of Ghana, Legon, Accra P.O. Box LG43, Ghana; kfopuni@ug.edu.gh; 3 Institute of Immunology, University Medicine Rostock, Schillingallee 69, 18059 Rostock, Germany; hj.thiesen@indymed.de; 4 Gesellschaft für Individualisierte Medizin mbH (IndyMed), Industriestrasse 15, 18069 Rostock, Germany

**Keywords:** SARS-CoV-2 Omicron, spike protein, receptor binding domain, receptor interaction, computational biology, transmissibility, disease severity

## Abstract

Our study focuses on free energy calculations of SARS-CoV-2 spike protein receptor binding motives (RBMs) from wild type and variants of concern (VOCs), with emphasis on SARS-CoV-2 Omicron. Our computational analysis underlines the occurrence of positive selection processes that specify Omicron host adaption and bring changes on the molecular level into context with clinically relevant observations. Our free energy calculation studies regarding the interaction of Omicron’s RBM with human angiotensin converting enzyme 2 (hACE2) indicate weaker binding to the receptor than Alpha’s or Delta’s RBMs. Upon weaker binding, fewer viruses are predicted to be generated in time per infected cell, resulting in a delayed induction of danger signals as a trade-off. Along with delayed immunogenicity and pathogenicity, more viruses may be produced in the upper respiratory tract, explaining enhanced transmissibility. Since in interdependence on the human leukocyte antigen type (HLA type), more SARS-CoV-2 Omicron viruses are assumed to be required to initiate inflammatory immune responses, and because of pre-existing partial immunity through previous infections and/or vaccinations, which mostly guard the lower respiratory tract, overall disease severity is expected to be reduced.

## 1. Amino Acid Sequence Alignments Point to a Shift in RBM Characteristics

**Figure 1:**
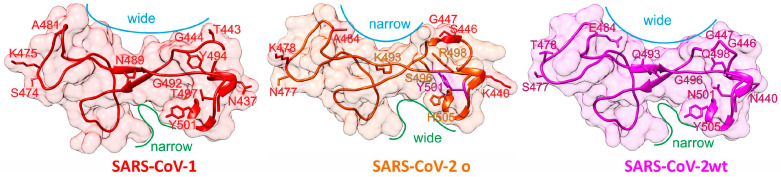
Structure comparisons of SARS-CoV receptor binding motives. Mutated amino acid residues in SARS-CoV-2 o and their counterparts in SARS-CoV-1 or SARS-CoV-2 wt (labeled) are shown as stick models. For further explanations see text.

Within the receptor binding domain (RBD; aa319 to aa541) of the wild type (wt) SARS-CoV-2 spike protein, the amino acid sequence stretch aa437 to aa508 encompasses the receptor binding motif (RBM) [1]. Amino acid residue exchanges have been observed at distinct RBM positions with all variants of concern (VOCs) [2]. The newly reported Omicron VOC carries ten exchanged amino acids (Figure 1) in its RBM, of which four (K440, S446, K478, and A484) are also found in SARS-CoV-1-, in bat-, and/or in civet-derived RBMs at the respective positions and through which Omicron’s RBM can be distinguished from that of SARS-CoV-2’s wt [3]. 

Figure 1 shows 3D structures of SARS-CoV-1 [4] and SARS-CoV-2 wt [1] from X-ray data, whereas the RBM structure of SARS-CoV-2 o has been modeled by AlphaFold [5]. Although the overall topology of the SARS-CoV-2 RBMs are quite comparable, structure details which result from distinct amino acid exchanges yield local surface differences, such as a narrower distance between A484 and G447 in Omicron’s RBM compared with that between E484 and G447 in wt’s RBM (blue lines in Figure 1). In contrast, the cleft which is located next to amino acid residue 505 is narrower in wt’s RBM as compared with that of Omicron (green lines in Figure 1). Both surface alterations indicate a somewhat different interaction geometry with the hACE2 receptor. From the remaining six exchanged amino acid residues, five (N477, K493, S496, R498, and H505) are unique to Omicron, which further differentiates Omicron’s from wt’s SARS-CoV-2 spike protein when comparing the here-assembled seven RBMs (Table 1). Importantly, Omicron encodes for Y501 which was found to strengthen binding in alpha, beta, and gamma VOCs [6]. Of note, bat RBMs (BM48-31 and Rp3) do not bind to hACE2 [7] whereas SARS-CoV-1 binds to hACE2 with lower affinity than does SARS-CoV-2 wt [8]. For assignments of virus variants and strains to SARS-CoV-2 phylogeny tree locations and phylogeography, see Supplement (Figure S1).

In detail: residue K478 has been designated the decisive amino acid exchange in Delta’s RBM [2]. K478 has been retained in Omicron, which, similar to residues K440, S446, and N477 (all three are rarely seen in other variants [9]), lends Omicron more “non-SARS-CoV-2 wt” characteristics, e.g., K478 matches with K465 in the RBM of SARS-CoV-1. When expressing A484, Omicron avoided the receptor binding weakening E484 residue found in alpha and in other VOCs [10]. The A484 matching residue from SARS-CoV-1’s RBM is A471 which is located adjacent to L472, one of the amino acid residues which is in direct contact with hACE2 and which has been assigned as important for species-specific binding [4]. Residue K493 in Omicron’s RBM is positioned where N479 is found in SARS-CoV-1’s RBM. This exchange in Omicron’s RBM was later corrected to R493. N479 of SARS-CoV-1’s RBM makes direct contact with hACE2 and is considered to be responsible for species-specific binding as well. An N479K exchange resulted in steric hindrance and in weakening of RBD-binding to hACE2 [11]. S496 and R498 are rare RBM mutations and adverse effects on binding can be estimated for R498 as opposed to Q498 on wt’s RBM because of charge repulsion [4]. Y501 was considered to strengthen binding to hACE2 considerably with respect to SARS-CoV-2 wt and was assumed to also increase virus replication rates [12,13,14]. Finally, H505 from SARS-CoV-2 Omicron is located where Y491 is placed in SARS-CoV-1. H505 replaces Y505 of SARS-CoV-2 wt’s RBD and of other VOCs, respectively [3]. Y505 is directly involved in binding to hACE2 and from the physicochemical properties of histidine vs. tyrosine one can conclude that the binding of Omicron’s RBD to hACE2 would not be positively affected by this exchange. 

In sum, because of the multitude of amino acid exchanges and because of slightly altered surface geometry, the interaction of Omicron’s RBM with hACE2 is assumed to be weaker than that of Alpha’s or Delta’s RBM with hACE2.

## 2. Free Energy Calculations Indicate Weaker Receptor Binding of Omicron’s RBM

To substantiate our hypothesis of weaker interactions between Omicron’s RBM and hACE2 as compared with those of other VOCs, we performed free energy difference calculations (ΔΔG calculations) [15,16] for the SARS-CoV-2-derived RBMs (wt vs. Alpha or Delta or Omicron) when bound to hACE2 (Table 2). We then compared these binding differences to respective RBM-hDPP-IV interactions [17]. Since human DPP-IV is considered not to function as a receptor for SARS-CoV-2 in vivo [7], calculations of free energy differences of RBM-hDPP-IV complexes served as controls. Of note, the MERS virus uses hDPP-IV as a receptor and SARS-CoV-2 wt has been assumed to as well being able to bind to hDPP-IV [18]. For a description of experimental procedures, see the Supplement.

According to ΔΔG calculations on the respective amino acid exchanges and their contributions to receptor binding, one observes that with respect to the wt RBM, the Alpha RBM achieved slightly stronger binding to hACE2 when summing up all amino acid residue energy differences which arise from the respective single amino acid exchanges. The Delta RBM neither gained nor lost binding strength compared with wt RBM-hACE2 binding. Surprisingly, the Omicron RBM-hACE2 complex is energetically less favored (ΔΔG: +4.41 kJ/mol) than the complex between wt RBM and hACE2 which means that Omicron’s RBM binding to hACE2 is weakened with respect to hACE2 binding of either the wt, the Alpha, or the Delta RBM. Notably, the presence of N417, though located outside Omicron’s RBM, is known to reduce hACE2 binding [12,18] which correlates with our calculations where N417 affords an increase in free binding energy compared with K417 of SARS-CoV-2 wt’s RBM (ΔΔG +0.49 kJ/mol); see Supplementary Table S1.

By contrast, ΔΔG value differences of RBD-hDPP-IV binding of all VOCs showed that all their respective complexes were bound to hDPP-IV with weaker forces than wt (Table 2). Of note, N417 weakens binding to hDPP-IV even more (ΔΔG +0.64 kJ/mol) than that to hACE2; see Supplementary Table S2. This stands in agreement with the observation that SARS-CoV-2 wt uses hACE2 as in vivo entry into host cells rather than hDPP-IV [4]. Interestingly, Omicron RBM binding with hDPP-IV requests a smaller increase in free energy (ΔΔG: +4.05 kJ/mol) than that of Omicron RBM binding with hACE2 (ΔΔG: +4.90 kJ/mol) when taking the K417N exchange into account. It remains to be investigated whether such a free energy difference is large enough to cause Omicron to switch host receptors in vivo, and hence, to possibly alter tropism and to eventually produce different disease symptoms.

As of yet, binding strength differences of SARS-CoV-2 VOCs’ spike proteins to hACE2 are estimated controversially, depending on molecular modelling and simulation approaches [19,20,21,22,23,24]. Experimental data on binding strengths of SARS-CoV-2 Omicron’s spike protein to hACE2 which so far have become available provide evidence that Omicron’s RBD interaction with hACE2 is weaker than that of Delta’s RBD [25,26,27], with one exception which states that binding strength of Delta’s or Omicron’s RBD to hACE2 was in similar ranges [28].

## 3. A Molecular Perspective on Transmissibility and Disease Outcome

Weaker binding of the spike protein to its receptor is assumed to slow down virus uptake into cells through cell surface membrane fusion and to direct virus uptake towards endocytosis-dependent cell entry. Less virus uptake per cell thus, elicits fewer danger signals, thereby retarding innate immune response [29] which over time might result in higher viral production in the upper respiratory tract. Also of interest, outside its RBM the Omicron spike protein carries the N679K, P681H, N679K, D614G exchanges [30,31] which may assist in enhancing the above mentioned mechanisms, and ultimately transmissibility [32,33]. Weaker RBM–hACE2 interaction stands in agreement with the postulated switch in Omicron’s cell entry mechanism towards a TMPRSS2-independent fusion and an associated major shift in replication properties [34], as efficient cell entry of viruses via endosomal uptake is not limited by receptor binding strength.

COVID-19 is considered a result of an overacting immune response mostly affecting the lower respiratory tract [35]. It is tempting to speculate whether Omicron’s assumed weaker RBM–hACE2 binding with respect to those of Alpha or Delta also contributed to clinical observations of less severe disease outcome upon SARS-CoV-2 Omicron infection compared with infections with other SARS-CoV-2 VOCs. A reduced entry/uptake of viral particles per cell and time which affects intracellular replication of SARS-CoV-2 in the lower respiratory tract will likely be associated with reduced and/or altered peptide loadings of HLA class I receptors and elicited CD8 T-cell mediated cytotoxicity. In parallel, reduced production of spike protein most likely leads to reduced presentation of antigen peptides by HLA class II on antigen presenting cells. Individuals with the same HLA type but altered peptide loads, e.g., because of virus variant-dependent proliferation differences, experience different signal strengths to the immune system. Different HLA-dependent signaling will also be observed between individuals who are infected with the same virus variant but are equipped with different HLA types which are present in respective ethnic populations [36]. Individual HLA polymorphisms seem to indeed have affected tropism and disease severity of COVID-19 [37]. According to our computational NetMHC predictions, a given HLA class I receptor configuration has greater impact on mitigating or enhancing disease severity as compared with virus strain variations (Table S3). Nevertheless, amino acid deletions, as exemplified by Omicron’s spike protein compared with wild type spike protein turn out to have stronger effects on differential HLA class peptide presentation (Table S4) than single point mutations (Table S5). 

Reports of disease severity upon infections with SARS-CoV-2 Omicron, though at first anecdotal [38] or based on preliminary clinical studies [39,40,41], were validated by recent clinical studies from other parts of the world [42,43], confirming an overall less severe, rather mild, or even asymptomatic disease outcome, which again stands in agreement with results from in vitro studies [44] as well as with weaker binding of the virus to its receptor. Enhanced replication of SARS-CoV-2 Omicron in nasal epithelial cells has been shown and supports higher contagiousness whereas the reduced viral yields in human lung cells are in line with reduced disease severity [45]. Both observations are in agreement with weaker RBM–hACE2 binding of SARS-CoV-2 Omicron, as pointed out above.

From the molecular perspectives outlined here, it seems plausible that SARS-CoV-2 Omicron fulfilled some key criteria of a host-adapted virus variant with high contagion potential and less severe disease outcome [46]. Particularly after monospecific vaccine administration, SARS-CoV-2 Omicron might challenge a human’s post-immunized waning antibody/B-cell responses to induce a more general and perhaps long-lasting immunity by extending protective antibody repertoires and by simultaneously enhancing T-cell mediated immunity, as reported for Delta [47] and very recently for Omicron as well [48], thereby ultimately preparing an individual to defeat more pathogenic SARS-CoV-2 variants in the future.

## Figures and Tables

**Table 1:** Amino acid sequence alignments of coronavirus spike protein receptor binding motifs.

Virus	Receptor Binding Motif/Amino Acid Sequence ^(a,b,c,d)^
SARS-2 wt	^437^ NSNNLDSKVGGNYNYLYRLFRKSNLKPFERDISTEIYQAGSTPCNGVEGFNCYFPLQSYGFQPTNGVGYQPY ^508^
SARS-2 α	^437^ NSNNLDSKVGGNYNYLYRLFRKSNLKPFERDISTEIYQAGSTPCNGV ** K ** GFNCYFPLQ ** P ** YGFQPT ** Y ** GVGYQPY ^508^
SARS-2 δ	^437^ NSNNLDSKVGGNYNY ** R ** YRLFRKSNLKPFERDISTEIYQAGS ** K ** PCNGVEGFNCYFPLQSYGFQPTNGVGYQPY ^508^
SARS-2 ο	^437^ **N** S**N** ** K ** **LD** SKV ** S ** **GNYNY** L**YR**L**FRK**SN**LKPFERDIS**TEIYQAG ** NK ** **PC** NGV**AG**F**NCY**F**PL** ** K ** **SY** ** S ** **F** ** R ** P**T** ** Y ** **GVG** ** H ** **QPY** ^508^
SARS-1 wt	^434^ **N** TRNI**D**ATST**GNYNY**K**YR**YL**R**HGK**L**R**PFERDIS**NVPFSPDG**KPC**TPP**A**-L**NCY**W**PL**ND**Y**G**F**YT**T**T**G**I**G**Y**QPY** ^504^
BM48-31	**N** T**N**S**LDS**--**S**NEFE**Y**R-**R**-**FR**HGKI**KP**YG**RD**L**S**NVLFNPSGGT**C**-SAE**G**L**NCY**K**PL**A**SY**G**F**TQSS**G**I**G**F**QPY**
Rp3	**N** TA**K**Q**D**QG-----Q**Y**Y**YR**SH**RK**TK**LKPFERD**L**S**SDE-NGV-RT-LS-----------T**Y**D**F**Y**P**SVP**V**AY**Q**AT

(a) Amino acid residues (single letter code) printed in red are realized exchanges in the respective SARS-CoV-2 VOC with respect to SARS-CoV-2 wt.; (b) amino acid residues printed in bold are found in SARS-CoV-2 Omicron’s RBM (shaded) as well as in SARS-CoV-1’s RBM and/or in bat/civet-derived RBMs.; (c) underlined residues are important for species-specific receptor binding.; (d) amino acid exchange Q493K was later corrected to Q493R.

**Table 2:** Spike protein receptor binding motif amino acid exchanges and changes of free energies with human ACE2 binding or human DPP-IV binding ^(a)^.

Amino Acid Residue Exchange	Variant of Concern and Human Binding Partner/Receptor Complex							
	Alpha ^(b,c)^			Delta ^(b,c)^			Omicron ^(b,c)^	
	hACE2	hDPP-IV		hACE2	hDPP-IV		hACE2	hDPP-IV
N440K	n.a.	n.a.		n.b.	n.a.		n.b.	n.b.
G446S	n.a.	n.a.		n.a.	n.b.		+1.05	n.b.
L452R	n.a.	n.a.		n.b.	n.b.		n.a.	n.a.
S477N	n.a.	n.a.		n.a.	n.a.		n.b.	+0.39
T478K	n.a.	n.a.		n.b.	+1.00		n.b.	+1.00
E484K/A ^(d)^	+0.07	−0.05		n.a.	n.a.		+0.10	−0.20
Q493K ^(e)^	n.a.	n.a.		n.a.	n.a.		+0.97	+0.83
S494P	n.b.	n.b.		n.a.	n.a.		n.a.	n.a.
G496S	n.a.	n.a.		n.a.	n.a.		+0.44	n.b.
Q498R	n.a.	n.a.		n.a.	n.a.		+0.84	n.b.
N501Y	−0.08	+0.19		n.a.	n.a.		−0.08	+0.19
Y505H	n.a.	n.a.		n.a.	n.a.		+1.09	+1.20
sum	−0.01	+0.14		±0.00	+1.00		+4.41	+3.41

(a) ΔΔG values in kJ/mol; red: exchange weakens binding; green: exchange strengthens binding; (b) n.a.: not applicable; amino acid residue exchange not realized; (c) n.b.: not binding; distance between atoms to other residues ≥ 5 Å; (d) realized in Omicron RBM; (e) later changed to Q493R, which reduces ΔΔG value differences by ca. 0.2 kJ/mol (see Supplementary Tables).

## Supplementary Materials

The following supporting information can be downloaded at: https://www.mdpi.com/article/10.3390/medicina58020226/s1, Methods; Figure S1: Phylogeny tree of SARS-CoV-2 strains; Table S1: Effects of SARS-CoV-2 RBD-exchanged amino acid residues on strengths of interaction with ACE2; Table S2: Effects of SARS-CoV-2 RBD-exchanged amino acid residues on strengths of interaction with DPP-IV; Table S3: Comparative Analysis of HLA class I peptides from spike proteins from Omicron variants and strains; Table S4: Determination of HLA peptide preferences depending on amino acids deleted in spike proteins from Omicron variants and strains; Table S5: HLA Peptide preferences depending on single point mutations in spike proteins of Omicron variants and strains.
