# Supplementary material for: From Free Binding Energy Calculations of SARS-CoV-2—Receptor Interactions to Cellular Immune Responses
Source: Medicina (Kaunas). 2022 Feb 2;58(2):226. doi: 10.3390/medicina58020226 (PMC8876798; doi:10.3390/medicina58020226)
Supplement: Supplementary file 1 [file medicina-58-00226-s001.zip › medicina-1548252-supplementary.pdf]

From Free Binding Energy Calculations of SARS-CoV-2 – Receptor Interactions to Cellular Immune Responses

Michael O. Glocker [1,*], Kwabena F.M. Opuni [2], and Hans-Juergen Thiesen [3,4]

[1] Proteome Center Rostock, University Medicine Rostock and University of Rostock, Schillingallee 69, 18059 Rostock, Germany
[2] Department of Pharmaceutical Chemistry, School of Pharmacy, College of Health Science, University of Ghana, P. O. Box LG43 Legon, Ghana
[3] Institute of Immunology, University Medicine Rostock, Schillingallee 70, 18059 Rostock, Germany
[4] Gesellschaft für Individualisierte Medizin mbH (IndyMed), Industriestrasse 15, 18069 Rostock, Germany

Supplement

[*]**Corresponding author**
Prof. Dr. Michael O. Glocker
Proteome Center Rostock
University Rostock Medical Center and Natural Science Faculty
University of Rostock
Schillingallee 69
18057 Rostock
Germany
Phone: +49 - 381 - 494 4930
FAX: +49 - 381 - 494 4932
e-mail: michael.glocker@med.uni-rostock.de
URL: https://pzr.med.uni-rostock.de

Methods

Visualization of 3D protein structures

The coordinates of all non-hydrogen atoms of SARS-CoV-1 wildtype (wt) RBM plus human angiotensin converting enzyme 2 (hACE2) receptor (PDB ID 2AJF) (1) were downloaded from the RCSB Protein Data Bank (www.rcsb.org). A new PDB file that contained only the atom coordinates of SARS-CoV-1 wt RBM was generated by editing the 2AJF PDB file using a word processing program and opening the PDB file as a text file. Then all of the lines that contained the coordinates of the atoms from the hACE2 receptor components were deleted. The process was repeated for the SARS-CoV-2 wt RBM plus hACE2 receptor (PDB ID 6MOJ; represents UniProt P0DTC2, Genbank MN908947.3, and EPI_ISL:402125) (2). The 3D structure of SARS-CoV-2 Omicron RBD was modeled by alphafold (https://colab.research.google.com/github/sokrypton/ColabFold/blob/main/AlphaFold2.ipynb#scrollTo=G4yBrceuFbf3) (3) using the amino acid sequences of the SARS-CoV-2 Omicron RBD (EPI_ISL_6640916) that was obtained from the GitHub homepage (https://github.com/cov-lineages/pango-designation/issues/343). Visualization of the protein structures was done using the UCSF Chimera (http://www.cgl.ucsf.edu/chimera/) molecular visualization software (4).

Calculation of binding energy changes of SARS-CoV-2-derived RBMs (wt vs. Alpha or Delta or Omicron) when bound to human ACE2 or human DPP-IV

The binding energy changes between the SARS-CoV-2 wt RBM (UniProt P0DTC2, Genbank MN908947.3, EPI_ISL:402125) and the hACE2 receptor complex upon site-specific exchanges of amino acid residues from the SARS-CoV-2 wt RBM were performed using the BeAtMuSiC web server (http://babylone.ulb.ac.be/beatmusic/query.php) (5). The PDB file of the SARS-CoV-2 wt RBM and hACE2 receptor complex (PDB ID: 6MOJ) served as the input file. The SARS-CoV-2 wt RBM and hACE2 receptor were selected as the two binding partners. The server automatically performed systematic mutations of interface residues of the atoms belonging to the SARS-CoV-2 wt RBM. The procedure was repeated using the SARS-CoV-2 wt RBM and hDPP-IV receptor complex. The SARS-CoV-2 wt RBM and hDPP-IV receptor complex was generated by replacing the hACE2 receptor of the SARS-CoV-2 wt RBM and hACE2 receptor complex (PDB ID: 6MOJ) with the hDPP-IV receptor from the MERS and hDPP-IV receptor complex (PDB ID: 4L72) using Chimera´s "matchmake" operation procedure.

HLA-binding prediction of SARS-CoV-2 Omicron variants

The spike protein amino acid sequences of five SARS-CoV-2 variants (EPI_ISL_402125, EPI_ISL_6640916, EPI_ISL_8414412, EPI_ISL_8449977, EPI_ISL_8546808) were split into 9-mer peptides. The binding affinities of these 9-mer peptides were subjected to analyses of binding with the 10 most abundant HLA-types using the NetMHC 4.0 Webservice: https://services.healthtech.dtu.dk/service.php?NetMHC-4.0 (6-9).

Literature References (Methods)

(1) Li F, Li W, Farzan M, Harrison SC. Structure of SARS Coronavirus Spike Receptor-Binding Domain Complexed with Receptor. Science 2005; 390: 1864-1868. doi: 10.1126/science.1116480

(2) 1. Lan, J.; Ge, J.; Yu, J.; Shan, S.; Zhou, H.; Fan, S.; Zhang, Q.; Shi, X.; Wang, Q.; Zhang, L.; et al. Structure of the SARS-CoV-2 spike receptor-binding domain bound to the ACE2 receptor. Nature 2020, 581, 215–221. https://doi.org/10.1038/s41586-020-2180-5.

(3) 5. Jumper, J.; Evans, R.; Pritzel, A.; Green, T.; Figurnov, M.; Ronneberger, O.; Tunyasuvunakool, K.; Bates, R.; Žídek, A.; Potapenko, A.; et al. Highly accurate protein structure prediction with AlphaFold. Nature 2021, 596, 583–589. https://doi.org/10.1038/s41586-021-03819-2.

(4) Pettersen, E.F.; Goddard, T.D.; Huang, C.C.; Couch, G.S.; Greenblatt, D.M.; Meng, E.C.; Ferrin, T.E. UCSF Chimera—A visualization system for exploratory research and analysis. J. Comput. Chem. 2004, 25, 1605–1612. https://doi.org/10.1002/jcc.20084

(5) Dehouck, Y.; Kwasigroch, J.M.; Rooman, M.; Gilis, D. BeAtMuSiC: Prediction of changes in protein–protein binding affinity on mutations. Nucleic Acids Res. 2013, 41, W333–W339, https://doi.org/10.1093/nar/gkt450.

(6) Andreatta M, Nielsen M. Gapped sequence alignment using artificial neural networks: application to the MHC class I system. Bioinformatics. 2016 Feb 15;32(4):511-7. Epub 2015 Oct 29.
(7) Lundegaard C, Lamberth K, Harndahl M, Buus S, Lund O, Nielsen M. NetMHC-3.0: accurate web accessible predictions of human, mouse and monkey MHC class I affinities for peptides of length 8-11. Nucleic Acids Res. 2008 Jul 1;36(Web Server issue):W509-12. Epub 2008 May 7.
(8) Buus S, Lauemøller SL, Worning P, Kesmir C, Frimurer T, Corbet S, Fomsgaard A, Hilden J, Holm A, Brunak S. Sensitive quantitative predictions of peptide-MHC binding by a 'Query by Committee' artificial neural network approach. Tissue Antigens. 2003 Nov;62(5):378-84.
(9) Nielsen M, Lundegaard C, Worning P, Lauemøller SL, Lamberth K, Buus S, Brunak S, Lund O. Reliable prediction of T-cell epitopes using neural networks with novel sequence representations. Protein Sci. 2003 May;12(5):1007-17.

Supplementary Figures

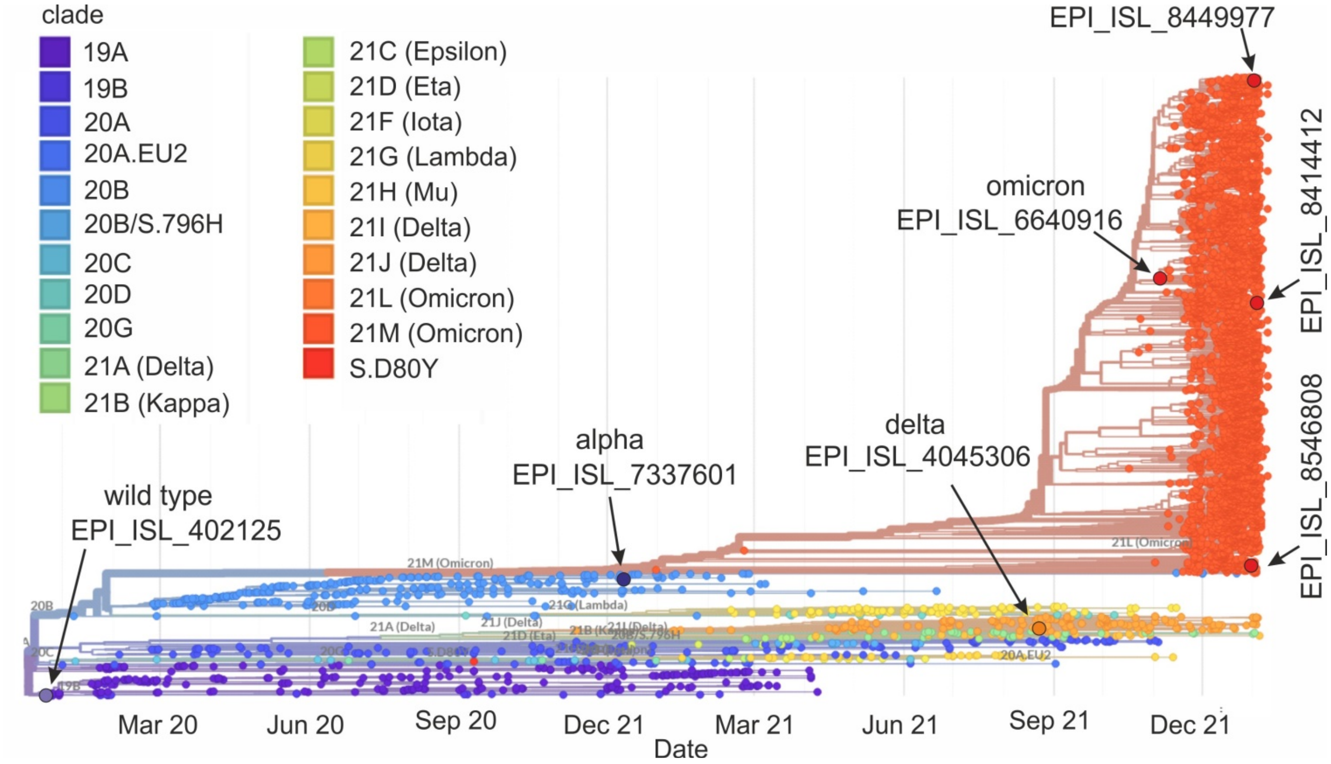

Figure S1. Phylogeny tree of SARS-CoV-2 strains. Clades are color coded. Locations of SARS-CoV-2 wt strain (EPI-ISL_402125) from Wuhan (GenBank: MN908947.3; spike protein UniProt P0DTC2), SARS-CoV-2 α strain from South Africa (EPI_ISL_7337601), SARS-CoV-2 ẟ strain from Germany (EPI_ISL_4045306), SARS-CoV-2 o strain from Botswana (EPI_ISL_6640916), SARS-CoV-2 o strain from Israel (EPI_ISL_8546808), SARS-CoV-2 o strain from Germany (EPI_ISL_8414412), and SARS-CoV-2 o strain from Singapore (EPI_ISL_8449977) are shown.

Obtained from: https://nextstrain.org/groups/neherlab/ncov/21K.Omicron?r=location

Supplementary Tables

Table S1: Effects of SARS-CoV-2 RBD-exchanged amino acid residues on strengths of interaction with hACE2. [a)]

	ΔΔG values in kcal / mol																			
amino acid residues in wt RBD [b)]	A	C	D	E	F	G	H	I	K	L	M	N	P	Q	R	S	T	V	W	Y
K417	+1.01	+0.83	+0.38	+0.51	+0.11	+0.74	+0.05	+0.28	±0.00	-0.00	-0.23	+0.49	+0.18	+0.47	+0.23	+0.87	+0.51	+0.79	-0.24	-0.10
G446	+1.27	+0.93	+1.05	+1.44	+1.17	±0.00	+0.89	+1.55	+1.22	+1.37	+1.33	+0.79	+1.49	+1.27	+1.13	+1.05	+1.32	+1.32	+1.27	+1.11
G447	+0.94	+0.92	+1.20	+1.40	+0.96	±0.00	+0.84	+1.01	+1.22	+1.09	+0.88	+1.03	+1.15	+1.09	+1.08	+1.01	+1.04	+1.08	+0.79	+0.97
Y449	+0.98	+1.04	+0.74	+0.95	+0.36	+0.89	+0.46	+0.63	+0.76	+0.59	+0.63	+0.64	+0.59	+0.83	+0.74	+0.84	+0.73	+0.65	+0.26	±0.00
Y453	+1.81	+0.42	+1.72	+1.54	+0.31	+1.95	+0.78	+0.64	+1.48	+0.76	+0.85	+1.42	+1.59	+1.40	+1.16	+1.65	+1.30	+0.71	+0.30	±0.00
L455	+1.62	+0.33	+2.09	+1.96	-0.14	+1.89	+0.61	+0.13	+1.69	±0.00	+0.55	+1.88	+1.06	+1.55	+0.93	+1.58	+1.31	+0.39	-0.07	-0.22
F456	+2.18	+1.49	+1.85	+1.94	±0.00	+2.45	+1.20	+1.09	+1.61	+1.25	+1.30	+1.83	+2.51	+1.68	+1.17	+2.12	+1.66	+1.32	+0.52	+0.57
Y473	+1.03	+0.87	+0.81	+1.04	+0.38	+1.17	+0.68	+0.58	+0.97	+0.62	+0.51	+0.82	+0.53	+0.87	+0.63	+0.92	+0.78	+0.76	+0.46	±0.00
A475	±0.00	+0.06	+1.42	+1.58	-0.32	+0.91	+0.45	+0.07	+1.07	+0.17	+0.19	+0.86	+0.63	+0.71	+0.74	+0.62	+0.54	+0.14	-0.30	-0.27
G476	+0.70	+0.30	+0.50	+0.81	+0.08	±0.00	+0.19	+0.44	+0.63	+0.47	+0.29	+0.37	+1.03	+0.59	+0.52	+0.50	+0.44	+0.40	+0.17	+0.23
E484	+0.10	-0.04	+0.07	±0.00	-0.09	+0.43	+0.07	-0.15	+0.07	+0.01	+0.09	+0.09	-0.43	+0.22	+0.10	+0.06	-0.04	-0.16	-0.12	-0.07
F486	+1.68	+1.60	+1.48	+1.34	±0.00	+1.89	+0.84	+0.90	+1.08	+0.74	+0.85	+1.33	+0.81	+1.20	+0.95	+1.46	+1.23	+0.93	+0.31	+0.50
N487	+1.13	+0.19	+0.97	+1.19	+0.02	+0.75	+0.18	+0.51	+1.08	+0.39	+0.25	±0.00	+1.16	+0.69	+0.60	+1.03	+0.80	+0.35	+0.23	-0.24
Y489	+2.82	+2.59	+2.79	+2.20	+1.38	+3.43	+1.74	+1.93	+1.98	+2.01	+1.68	+2.58	+2.84	+1.97	+1.85	+2.87	+2.47	+2.36	+0.84	±0.00
Q493	+1.52	+0.83	+1.46	+1.36	+0.10	+1.84	+0.42	+0.56	+0.97	+0.45	+0.57	+1.15	+1.36	±0.00	+0.60	+2.02	+1.10	+0.71	-0.08	+0.15
G496	+0.63	+0.02	+0.80	+1.05	+0.15	±0.00	+0.33	+0.41	+1.12	+0.58	+0.40	+0.67	+1.11	+0.78	+0.71	+0.44	+0.41	+0.22	+0.18	+0.21
Q498	+0.75	-0.38	+1.12	+1.64	-0.27	+0.98	+0.26	-0.37	+1.46	-0.49	-0.28	+0.80	+1.26	±0.00	+0.84	+0.74	+0.19	-0.01	-0.25	-0.26
T500	+1.62	+1.57	+1.08	+1.93	+0.36	+2.30	+0.91	+0.80	+1.74	+0.40	+0.46	+1.01	+1.75	+1.47	+0.99	+1.38	±0.00	+0.83	+0.05	+0.09
N501	+0.88	+0.46	+0.80	+0.77	-0.09	+1.24	+0.33	+0.23	+0.46	+0.06	-0.18	±0.00	+1.19	+0.56	+0.43	+1.25	+0.42	+0.20	-0.08	-0.08
G502	+1.21	+0.91	+1.38	+2.16	-0.51	±0.00	+0.04	+0.87	+1.24	+0.51	+0.41	+1.05	+0.97	+1.20	+0.65	+1.23	+1.09	+0.99	-0.49	-0.28
V503	+0.34	+0.42	+0.08	+0.42	+0.45	+0.41	+0.16	+0.29	+0.39	+0.26	+0.28	+0.12	+0.04	+0.36	+0.17	+0.09	+0.11	±0.00	+0.14	+0.44
Y505	+1.78	+1.83	+1.74	+1.85	+0.86	+2.51	+1.09	+1.45	+1.82	+1.24	+1.14	+1.54	+1.94	+1.24	+1.09	+1.80	+1.71	+1.95	+0.13	±0.00

a) no cumulative effects; free energy difference calculations for individual amino acid positions.

b) residues with a shortest atom distance below 5 Å between RBD and hACE2.

Table S2: Effects of SARS-CoV-2 RBD-exchanged amino acid residues on strengths of interaction with hDPP-IV. [a)]

	ΔΔG values in kcal / mol																			
amino acid residues in wt RBD [b)]	A	C	D	E	F	G	H	I	K	L	M	N	P	Q	R	S	T	V	W	Y
R403	+0.77	+0.31	+0.67	+0.58	+0.20	+0.99	+0.02	+0.24	+0.58	+0.31	+0.29	+0.63	+0.25	+0.49	±0.00	+0.64	+0.38	+0.53	+0.11	+0.31
D405	+0.43	+0.45	±0.00	+0.31	-0.10	+0.57	-0.06	+.025	+0.32	+0.26	+0.20	+0.08	+0.27	+0.20	+0.26	+0.21	+0.23	+0.43	-0.14	+0.05
E406	+0.07	+0.69	+0.64	±0.00	+0.02	+0.78	+0.08	+0.50	+0.72	+0.27	+0.16	+0.43	+1.13	+0.47	+0.38	+0.67	+0.61	+0.39	+0.19	-0.01
R408	+0.53	+0.35	+0.11	+0.19	-0.11	+0.51	-0.13	+0.46	+0.16	+0.07	+0.03	-0.04	+0.11	+0.18	±0.00	+0.23	+0.13	+0.73	+0.17	+0.12
Q409	+1.05	+0.64	+0.64	+0.62	+0.06	+0.66	+0.11	+0.23	+0.47	+0.39	+0.38	+0.48	+0.81	±0.00	+0.47	+0.64	+0.67	+0.55	+0.23	+0.07
T415	+1.03	+0.83	+0.91	+1.21	+0.58	+1.15	+0.46	+0.66	+0.98	+0.70	+0.85	+0.75	+1.13	+0.76	+0.71	+0.73	±0.00	+0.60	+0.59	+0.69
G416	+0.75	+0.34	+0.74	+1.49	+0.44	±0.00	+0.46	+0.58	+1.38	+0.68	+0.55	+1.03	+1.02	+1.15	+0.73	+0.76	+0.84	+0.74	+0.42	+0.38
K417	+1.44	+0.49	+0.69	+0.44	-0.32	+0.92	+0.02	+0.15	±0.00	+0.07	+0.10	+0.64	+0.45	+0.50	+0.46	+1.23	+0.91	+0.90	-0.34	-0.18
L455	+1.17	+0.56	+1.30	+1.16	+0.37	+1.54	+0.64	+0.40	+0.99	±0.00	+0.61	+1.26	+0.74	+0.91	+1.04	+1.04	+0.91	+0.54	+0.56	+0.39
F456	+2.41	+1.46	+2.15	+2.31	±0.00	+2.56	+1.42	+1.09	+1.90	+1.09	+1.39	+1.84	+2.50	+1.85	+1.65	+2.19	+1.74	+1.24	+0.60	+0.75
I472	+1.07	+0.64	+1.28	+1.30	+0.29	+1.12	+0.69	±0.00	+1.08	+0.32	+0.55	+0.96	+0.51	+1.10	+0.90	+1.06	+0.77	+0.41	+0.40	+0.53
Y473	+1.33	+1.00	+1.31	+1.48	+0.41	+1.50	+0.88	+0.55	+1.22	+0.63	+0.53	+1.17	+0.90	+1.15	+0.87	+1.24	+0.94	+0.63	+0.51	±0.00
Q474	+0.14	-0.01	+0.20	+0.38	-0.58	+0.11	-0.26	-0.35	+0.27	-0.53	-0.12	+0.16	-0.27	±0.00	-0.18	+0.19	+0.18	-027	-0.37	-0.36
A475	±0.00	-0.12	+1.93	+1.89	-1.14	+1.15	+0.25	-0.05	+1.07	-0.09	-0.23	+1.35	+1.20	+1.10	+0.56	+1.05	+0.91	+0.30	-1.26	-1.14
G476	+0.58	-0.88	+0.75	+1.40	-1.61	±0.00	-0.81	-0.69	+1.16	-0.41	-0.59	+0.40	+1.11	+0.63	+0.14	+0.22	+0.23	-0.51	-0.99	-0.98
S477	+0.14	-0.03	+0.36	+0.59	+0.32	+0.33	+0.28	+0.20	+0.56	+0.21	+0.21	+0.39	-0.11	+0.42	+0.47	±0.00	+0.13	+0.15	+0.36	+0.36
T478	+0.31	+0.28	+0.55	+1.05	+0.15	+0.60	+0.16	+0.02	+1.00	-0.02	+0.08	+0.44	+0.36	+0.65	+0.69	+0.17	±0.00	-0.14	+0.51	+0.38
P479	+0.25	+0.35	+0.19	+0.41	+0.51	+0.50	+0.30	+0.26	+0.41	+0.29	+0.38	+0.26	±0.00	+0.37	+0.36	+0.14	+0.14	+0.25	+0.50	+0.38
C480	+0.38	±0.00	+0.60	+0.67	+0.14	+0.89	+0.43	+0.16	0.49	+0.17	+0.26	+0.71	+0.46	+0.53	+0.47	+0.36	+0.43	+0.33	+0.13	+0.08
V483	+0.41	+0.16	+0.53	+0.24	+0.31	+0.54	+0.23	+0.05	+0.14	+0.20	+0.17	+0.36	+0.87	+0.11	+0.19	+0.37	+0.15	±0.00	+0.44	+0.35
E484	-0.20	-0.21	0.06	±0.00	-0.32	+0.36	-0.08	-0.34	-0.05	-0.40	-0.22	-0.02	-0.22	-0.03	-0.11	0.00	-0.01	-0.25	-0.27	-0.22
G485	+1.24	+0.45	+0.83	+1.28	-0.00	±0.00	+0.45	+0.45	+0.80	+0.33	+0.10	+0.19	+0.53	+0.98	+0.52	+0.86	+0.60	+0.41	+0.24	+0.50
F486	+4.18	+3.34	+3.45	+3.20	±0.00	+4.41	+2.05	+2.51	+3.48	+2.21	+2.09	+3.26	+3.46	+2.88	+2.50	+3.90	+3.24	+3.13	+0.87	+0.82
N487	+0.42	-1.78	+1.39	+2.62	-2.27	-0.12	-0.83	-1.11	+2.05	-1.06	-1.38	±0.00	+0.88	+0.74	+0.10	+0.39	+0.40	-0.89	-2.03	-2.25
C488	+2.03	±0.00	+2.49	+2.74	+0.30	+2.23	+1.19	+1.06	+2.25	+0.99	+0.83	+2.08	+3.53	+1.55	+1.53	+1.90	+2.02	+1.33	+0.46	+0.40
Y489	+2.37	+2.04	+2.53	+1.97	+0.81	+2.76	+1.32	+1.49	+1.74	1.75	+1.33	+2.15	+2.53	+1.71	+1.41	+2.45	+2.16	+1.90	+0.58	±0.00
Q493	+0.71	+52	0.84	+0.80	-0.13	+0.98	+0.27	+0.15	+0.83	+0.04	+0.31	+0.58	+0.87	±0.00	+0.63	+1.02	+0.47	+0.22	-0.07	+0.11
T500	+0.17	-0.02	-0.15	+0.12	+0.19	+0.26	+0.10	+0.26	+0.12	+0.14	+0.17	-0.09	+0.33	+0.20	+0.18	+0.03	±0.00	+0.26	+0.15	+0.15
N501	+0.67	+0.73	+0.67	+0.38	+0.30	+0.84	+0.02	+0.44	+0.21	+0.43	+0.35	±0.00	+0.68	+0.21	+0.18	+0.79	+0.09	+0.46	+0.25	+0.19
G502	+1.59	+0.92	+1.91	+2.50	+0.19	±0.00	+0.55	+1.27	+2.13	+0.88	+0.91	+1.68	+1.15	+1.89	+1.44	+1.50	+1.38	+1.37	+0.07	+0.46
V503	+0.27	+0.46	+0.01	+0.37	+0.48	+0.42	+0.10	+0.23	+0.25	+0.17	+0.18	+0.05	-0.17	+0.24	+0.02	+0.10	+0.05	±0.00	+0.25	+0.36
Y505	+1.85	+2.07	+1.71	+1.67	+0.71	+2.35	+1.20	+1.45	+1.72	+1.11	+0.94	+1.41	+1.94	+1.20	+1.32	+1.70	+1.64	+1.57	+0.48	±0.00

a) no cumulative effects; free energy difference calculations for individual amino acid positions.

b) residues with a shortest atom distance below 5 Å between RBD and hDPP-IV.

Table S3: Comparative Analysis of HLA class I peptides from spike proteins from Omicron variants and strains. [a,b,c)]

	HLA-A0101	HLA-A0201	HLA-A0301	HLA-A2402	HLA-B0702	HLA-B0801	HLA-B1503	HLA-B3501	HLA-B4001	HLA-B4402
**Strong Binder**										
EPI_ISL_402125	2	13	5	4	2	2	41	18	2	1
EPI_ISL_6640916	2	12	5	4	2	2	38	17	2	1
EPI_ISL_8414412	3	11	6	5	1	2	38	18	2	1
EPI_ISL_8449977	3	11	6	5	1	2	38	18	2	1
EPI_ISL_8546808	2	12	5	4	1	2	41	18	2	1
**Medium Binder**										
EPI_ISL_402125	4	25	18	10	10	15	94	34	5	4
EPI_ISL_6640916	4	27	18	11	7	15	100	35	4	3
EPI_ISL_8414412	3	26	18	9	8	16	102	33	4	3
EPI_ISL_8449977	3	26	18	9	8	16	101	33	4	3
EPI_ISL_8546808	3	24	18	9	9	15	96	33	5	4
**Nonbinder**										
EPI_ISL_402125	1259	1227	1242	1251	1253	1248	1130	1213	1258	1260
EPI_ISL_6640916	1256	1223	1239	1247	1253	1245	1124	1210	1256	1258
EPI_ISL_8414412	1253	1222	1235	1245	1250	1241	1119	1208	1253	1255
EPI_ISL_8449977	1253	1222	1235	1245	1250	1241	1120	1208	1253	1255
EPI_ISL_8546808	1255	1224	1237	1247	1250	1243	1123	1209	1253	1255

a) numbers of peptides with high, medium, or no binding affinity are listed as determined by the NetMHC 4.0 Server.

b) HLA-B1503 has the highest number of strong and/or medium binding peptides.

c) the lowest number of strong binding peptides is present in HLA-B4402.

Table S4: Determination of HLA peptide preferences depending on amino acids deleted in spike proteins from Omicron variants and strains. [a,b)]

pos	EPI_ISL_402125	EPI_ISL_6640916	EPI_ISL_8414412	EPI_ISL_8449977	EPI_ISL_8546808		amino acid sequence	EPI_ISL_402125		EPI_ISL_6640916		EPI_ISL_8414412		EPI_ISL_8449977		EPI_ISL_8546808		
62	V	V	V	V	V	*	FFSNVTWFH	0,280	HLA-B3501	0,280	HLA-B3501	0,280	HLA-B3501	0,280	HLA-B3501	0,280	HLA-B3501	Deletion Region 1
63	T	T	T	T	T	*	FSNVTWFHA	0,350	HLA-A0101	0,350	HLA-A0101	0,415	HLA-A0201	0,415	HLA-A0201	0,350	HLA-A0101	
64	W	W	W	W	W	*	SNVTWFHAI	0,279	HLA-B1503	0,279	HLA-B1503	0,324	HLA-A2402	0,324	HLA-A2402	0,279	HLA-B1503	
65	F	F	F	F	F	*	NVTWFHAIH	0,251	HLA-B3501	0,251	HLA-B3501	0,143	HLA-B3501	0,143	HLA-B3501	0,149	HLA-B3501	
66	H	H	H	H	H	*	VTWFHAIHV	0,509	HLA-A0201	0,509	HLA-A0201	0,214	HLA-B1503	0,214	HLA-B1503	0,229	HLA-B1503	
67	A	A	V	V	A	.	TWFHAIHVS	0,181	HLA-A2402	0,181	HLA-A2402	0,088	HLA-A0201	0,088	HLA-A0201	0,088	HLA-A2402	
68	I	I	I	I	I	*	WFHAIHVSG	0,152	HLA-B0801	0,152	HLA-B0801	0,126	HLA-B3501	0,126	HLA-B3501	0,168	HLA-B3501	
69	H	H	-	-	-		FHAIHVSGT	0,100	HLA-B1503	0,100	HLA-B1503							
70	V	V	-	-	-		HAIHVSGTN	0,389	HLA-B3501	0,389	HLA-B3501							
71	S	S	S	S	S	*	AIHVSGTNG	0,115	HLA-B1503	0,115	HLA-B1503	0,164	HLA-B1503	0,164	HLA-B1503	0,133	HLA-B1503	
72	G	G	G	G	G	*	IHVSGTNGT	0,084	HLA-B1503	0,084	HLA-B1503	0,284	HLA-B3501	0,284	HLA-B3501	0,413	HLA-B3501	
73	T	T	T	T	T	*	HVSGTNGTK	0,477	HLA-A0301	0,477	HLA-A0301	0,489	HLA-A0301	0,489	HLA-A0301	0,501	HLA-A0301	
74	N	N	N	N	N	*	VSGTNGTKR	0,120	HLA-A0301	0,120	HLA-A0301	0,135	HLA-A0301	0,135	HLA-A0301	0,135	HLA-A0301	
75	G	G	G	G	G	*	SGTNGTKRF	0,365	HLA-B1503	0,365	HLA-B1503	0,365	HLA-B1503	0,365	HLA-B1503	0,365	HLA-B1503	
76	T	T	T	T	T	*	GTNGTKRFD	0,047	HLA-B4001	0,047	HLA-B4001	0,047	HLA-B4001	0,047	HLA-B4001	0,047	HLA-B4001	
137	N	N	N	N	N	*	FQFCNDPFL	0,795	HLA-A0201	0,795	HLA-A0201	0,795	HLA-A0201	0,795	HLA-A0201	0,795	HLA-A0201	Deletion Region 2
138	D	D	D	D	D	*	QFCNDPFLG	0,099	HLA-A2402	0,085	HLA-A2402	0,085	HLA-A2402	0,085	HLA-A2402	0,085	HLA-A2402	
139	P	P	P	P	P	*	FCNDPFLGV	0,374	HLA-A0201	0,265	HLA-A0201	0,198	HLA-B3501	0,198	HLA-B3501	0,198	HLA-B3501	
140	F	F	F	F	F	*	CNDPFLGVY	0,476	HLA-A0101	0,492	HLA-A0101	0,090	HLA-A0301	0,090	HLA-A0301	0,090	HLA-A0301	
141	L	L	L	L	L	*	NDPFLGVYY	0,144	HLA-B4402	0,167	HLA-B3501	0,041	HLA-B3501	0,041	HLA-B3501	0,041	HLA-B3501	
142	G	D	-	-	-		DPFLGVYYH	0,483	HLA-B3501	0,446	HLA-B3501							
143	V	V	-	-	-		PFLGVYYHK	0,145	HLA-A0301	0,114	HLA-A0301							
144	Y	Y	-	-	-		FLGVYYHKN	0,157	HLA-B0801	0,172	HLA-A0101							
145	Y	Y	D	D	D		LGVYYHKNN	0,084	HLA-B1503	0,058	HLA-B1503	0,132	HLA-B3501	0,132	HLA-B3501	0,132	HLA-B3501	
146	H	H	H	H	H	*	GVYYHKNNK	0,699	HLA-A0301	0,371	HLA-A0301	0,089	HLA-A0301	0,089	HLA-A0301	0,089	HLA-A0301	
147	K	K	K	K	K	*	VYYHKNNKS	0,128	HLA-B1503	0,128	HLA-B1503	0,188	HLA-A0201	0,188	HLA-A0201	0,188	HLA-A0201	
148	N	N	N	N	N	*	YYHKNNKSW	0,500	HLA-A2402	0,500	HLA-A2402	0,210	HLA-B4402	0,210	HLA-B4402	0,210	HLA-B4402	
149	N	N	N	N	N	*	YHKNNKSWM	0,337	HLA-B1503	0,337	HLA-B1503	0,110	HLA-B1503	0,110	HLA-B1503	0,110	HLA-B1503	

Table S4 (continued)

pos	EPI_ISL_402125	EPI_ISL_6640916	EPI_ISL_8414412	EPI_ISL_8449977	EPI_ISL_8546808		amino acid sequence	EPI_ISL_402125		EPI_ISL_6640916		EPI_ISL_8414412		EPI_ISL_8449977		EPI_ISL_8546808		
208	T	T	T	T	T	*	YSKHTPINL	0,546	HLA-B1503	0,546	HLA-B1503	0,488	HLA-B1503	0,488	HLA-B1503	0,546	HLA-B1503	Deletion Region 3
209	P	P	P	P	P	*	SKHTPINLV	0,475	HLA-B1503	0,245	HLA-B1503	0,295	HLA-B1503	0,295	HLA-B1503	0,475	HLA-B1503	
210	I	I	I	I	I	*	KHTPINLVR	0,131	HLA-A0301	0,119	HLA-A0301	0,046	HLA-B0702	0,046	HLA-B0702	0,131	HLA-A0301	
211	N	N	-	-	N		HTPINLVRD	0,052	HLA-B4001	0,052	HLA-B4001					0,052	HLA-B4001	
212	L	L	I	I	L	:	TPINLVRDL	0,509	HLA-B0702	0,656	HLA-B0702	0,092	HLA-A0201	0,092	HLA-A0201	0,509	HLA-B0702	
213	V	G	V	V	V		PINLVRDLP	0,054	HLA-A0101	0,046	HLA-A0101	0,161	HLA-B3501	0,161	HLA-B3501	0,054	HLA-A0101	
214	R	R	R	R	R	*	INLVRDLPQ	0,117	HLA-B1503	0,085	HLA-B0801	0,075	HLA-B1503	0,075	HLA-B1503	0,117	HLA-B1503	
215	D	D	D	D	D	*	NLVRDLPQG	0,085	HLA-A0201	0,075	HLA-A0201	0,067	HLA-B3501	0,067	HLA-B3501	0,085	HLA-A0201	
216	L	L	L	L	L	*	LVRDLPQGF	0,480	HLA-B1503	0,532	HLA-B1503	0,448	HLA-B1503	0,448	HLA-B1503	0,480	HLA-B1503	
217	P	P	P	P	P	*	VRDLPQGFS	0,036	HLA-A0101	0,033	HLA-B4001	0,036	HLA-A0101	0,036	HLA-A0101	0,036	HLA-A0101	
218	Q	Q	Q	Q	Q	*	RDLPQGFSA	0,226	HLA-B1503	0,226	HLA-B1503	0,226	HLA-B1503	0,226	HLA-B1503	0,226	HLA-B1503	

a) single point mutations (yellow) and deletions (red) are highlighted in the first 6 columns

b) binding affinity is color coded yelow (medium: 0.43 – 0.64) or green (strong: >0.64)

Table S5: HLA Peptide preferences depending on single point mutations in spike proteins of Omicron variants and strains. [a,b,c,d)]

Pos	EPI_ISL_402125	EPI_ISL_6640916	EPI_ISL_8414412	EPI_ISL_8449977	EPI_ISL_8546808		amino acid sequence	EPI_ISL_402125		EPI_ISL_6640916		EPI_ISL_8414412		EPI_ISL_8449977		EPI_ISL_8546808	
501	N	Y	Y	Y	N		FQPTNGVGY	0,753	HLA-B1503	0,169	HLA-B1503	0,169	HLA-B1503	0,169	HLA-B1503	0,753	HLA-B1503
684	A	A	A	A	A	*	SPRRARSVA	0,867	HLA-B0702	0,231	HLA-B0702	0,231	HLA-B0702	0,231	HLA-B0702	0,231	HLA-B0702
496	G	G	S	S	G	.	LQSYGFQPT	0,717	HLA-B1503	0,235	HLA-B1503	0,376	HLA-B1503	0,376	HLA-B1503	0,717	HLA-B1503
146	H	H	H	H	H	*	GVYYHKNNK	0,699	HLA-A0301	0,371	HLA-A0301	0,089	HLA-A0301	0,089	HLA-A0301	0,089	HLA-A0301
491	P	P	P	P	P	*	NCYFPLQSY	0,645	HLA-B1503	0,498	HLA-B1503	0,498	HLA-B1503	0,498	HLA-B1503	0,645	HLA-B1503
421	Y	Y	Y	Y	Y	*	KIADYNYKL	0,669	HLA-A0201	0,539	HLA-A0201	0,539	HLA-A0201	0,539	HLA-A0201	0,669	HLA-A0201
338	F	F	F	F	F	*	NLCPFGEVF	0,671	HLA-B1503	0,574	HLA-B1503	0,574	HLA-B1503	0,574	HLA-B1503	0,671	HLA-B1503
509	R	R	R	R	R	*	YQPYRVVVL	0,691	HLA-B1503	0,599	HLA-B1503	0,599	HLA-B1503	0,599	HLA-B1503	0,691	HLA-B1503
412	P	P	P	P	P	*	RQIAPGQTG	0,713	HLA-B1503	0,602	HLA-B1503	0,713	HLA-B1503	0,713	HLA-B1503	0,713	HLA-B1503
376	T	A	T	T	T	:	ASFSTFKCY	0,755	HLA-B1503	0,634	HLA-B3501	0,621	HLA-B3501	0,621	HLA-B3501	0,755	HLA-B1503
856	N	N	K	K	K	:	AQKFNGLTV	0,668	HLA-B1503	0,668	HLA-B1503	0,618	HLA-B1503	0,618	HLA-B1503	0,618	HLA-B1503
980	I	I	I	I	I	*	VLNDILSRL	0,675	HLA-A0201	0,675	HLA-A0201	0,620	HLA-A0201	0,620	HLA-A0201	0,620	HLA-A0201
798	G	G	G	G	G	*	IKDFGGFNF	0,592	HLA-B1503	0,836	HLA-B1503	0,836	HLA-B1503	0,836	HLA-B1503	0,836	HLA-B1503
371	S	F	L	L	S		VLYNSASFS	0,425	HLA-B1503	0,838	HLA-B1503	0,795	HLA-B1503	0,795	HLA-B1503	0,425	HLA-B1503
497	F	F	F	F	F	*	QSYGFQPTN	0,160	HLA-B1503	0,815	HLA-B1503	0,872	HLA-B1503	0,872	HLA-B1503	0,160	HLA-B1503
447	G	G	G	G	G	*	SKVGGNYNY	0,623	HLA-B1503	0,623	HLA-B1503	0,767	HLA-B1503	0,767	HLA-B1503	0,623	HLA-B1503
374	F	F	F	F	F	*	NSASFSTFK	0,576	HLA-A0301	0,325	HLA-A0301	0,680	HLA-A0301	0,680	HLA-A0301	0,576	HLA-A0301
207	H	H	H	H	H	*	IYSKHTPIN	0,242	HLA-A2402	0,242	HLA-A2402	0,675	HLA-A2402	0,675	HLA-A2402	0,242	HLA-A2402
343	N	N	N	N	N	*	GEVFNATRF	0,635	HLA-B4001	0,265	HLA-B4402	0,265	HLA-B4402	0,265	HLA-B4402	0,635	HLA-B4001

a) predictions calculated online with NetMHC 4.0

b) peptide-lengths: 9mer

c) single point mutations as compared to wild type (EPI_ISL_402125) are highlighted in the first 6 columns

d) binding affinity is color coded yellow (medium: 0.43-0.64) or green (strong: >0.64)
